# Supplementary material for: College Classroom Instructors Can Effectively Promote Standing among Students Provided with Standing Desks
Source: Int J Environ Res Public Health. 2021 Apr 22;18(9):4464. doi: 10.3390/ijerph18094464 (PMC8122831; doi:10.3390/ijerph18094464)
Supplement: Supplementary file 1 [file ijerph-18-04464-s001.zip › ijerph-1162071-file S1-final.pdf]

Desk #________ ActivPAL # _________ Group # ________

Stand-U Study II - Participant questionnaire

Instructions: Please complete the questions below. You may leave any question blank if you do not want to answer it.

1. What is your age: ____________years

2. What is your gender: (circle one)

Male	Female	Other: _____________(specify)

3. What grade are you in: (circle one)

Freshman	Sophomore	Junior
Senior	Graduate student	Other: _______________ (specify)

4. What is your race/ethnicity: (circle one)

White/Caucasian	Hispanic/Latinx	American Indian or Alaska native	Other: __________ (specify)
Black/African American	Asian descent	Native Hawaiian or other Pacific Islander	

5. What is your:

Height		Weight
_________ feet	_________inches	___________ lbs.

6. Did you stand at the standing desk? (circle one)

Yes	No

7. **IF YES TO #6**, For which reasons did you stand at the standing desk? **Please check all that apply.**

☐ To improve health	☐ Standing desks are new and cool	☐ Others are standing	☐ To reduce back pain
☐ I prefer standing to sitting	☐ Standing helps me learn better in class	☐ Encouraged by instructor to stand	☐ Helps reduce phone/laptop distractions
☐ To break up sitting time	☐ Increases attention/focus	☐ Can see the instructor/front of room better	☐ Makes me feel more accountable in class

Other reason(s): ____

____

8. **IF NO TO 6**, Why did you NOT stand at the standing desk? **Please check all that apply.**

☐ Too tired	☐ Standing would distract others	☐ No one else is standing	☐ Standing would block others' view
☐ I prefer sitting to standing	☐ Unable to use laptop/phone while standing	☐ No encouragement to stand	☐ Unable to stand due to injury or other health reason(s)
☐ Do not want to be seen standing	☐ Standing feels awkward socially	☐ Desk is too short or tall	☐ Cultural or religious reasons

Other reason(s): ____

____

9. In general, what are barriers or challenges for students using standing desks in a college classroom? **Please check all that apply.**

☐ Being tired	☐ Don't want to distract others	☐ No one else is standing	☐ Cultural or religious reasons
☐ Unable to stand due to injury or health reason(s)	☐ It is not comfortable to stand	☐ Did not know how to adjust the desk to be able to stand	☐ No encouragement to stand
☐ Desk is too short or tall	☐ Desktop is too small	☐ Don't want to invade neighbors' space	☐ Don't want to be only one standing

Other barrier(s): ____

____

10. In your opinion, what would be an optimal amount of time to spend standing in class?

___________ **minutes per 60 minute class**

11. Were the number of prompts to stand: (circle one)

Too many	Just right	Not enough	Did not see any prompts

The last questions are about the time you spend sitting while at work, at home, while doing course work and during leisure time. This may include time spent sitting at a desk, visiting friends, reading or sitting or lying down to watch television.

12. During the last 7 days, how much time did you usually spend **sitting** on a **weekday?**
_______ **hours per day**
_______ **minutes per day**

13. During the last 7 days, how much time did you usually spend **sitting** on a **weekend day?**
_______ **hours per day**
_______ **minutes per day**

Thank you for taking our survey!
