# Supplementary figures and images for: College Classroom Instructors Can Effectively Promote Standing among Students Provided with Standing Desks
Source: Int J Environ Res Public Health. 2021 Apr 22;18(9):4464. doi: 10.3390/ijerph18094464 (PMC8122831; doi:10.3390/ijerph18094464)

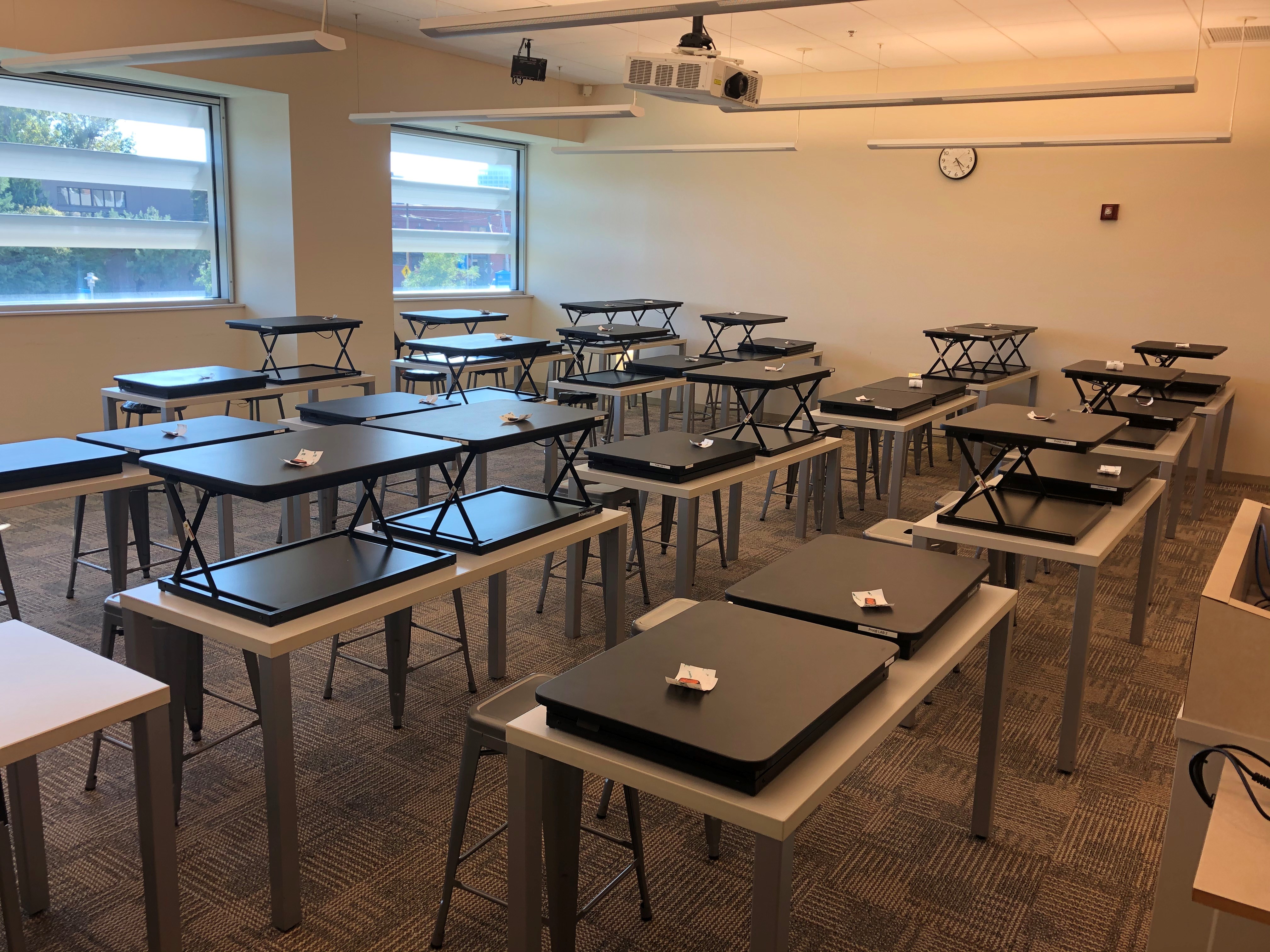

Supplement: Supplementary file 1 [file ijerph-18-04464-s001.zip › ijerph-1162071-figure S1-final.jpg]
